# Stray dogs in Nepal have high prevalence of vector-borne pathogens: a molecular survey

**DOI:** 10.1186/s13071-020-04057-7

**Published:** 2020-04-21

**Authors:** David Díaz-Regañón, Beatriz Agulla, Bidur Piya, Natalia Fernández-Ruiz, Alejandra Villaescusa, Mercedes García-Sancho, Fernando Rodríguez-Franco, Ángel Sainz

**Affiliations:** 1 Department of Animal Medicine and Surgery, College of Veterinary Medicine, Complutense University of Madrid, Avda. Puerta de Hierro s/n, 28040 Madrid, Spain; 2 Kathmandu Animal Treatment (KAT) Centre, Chapali Gaon, Budanilkantha, GPO Box 8975, EPC 4120 Kathmandu, Nepal; 3 Department of Animal Pathology, Faculty of Veterinary Medicine, University of Zaragoza, Calle de Miguel Servet 177, 50013 Zaragoza, Spain

**Keywords:** Nepal, Canine vector borne disease, PCR, FTA card, *Leishmania*, *Ehrlichia*, *Anaplasma*, *Babesia*, *Theileria*, *Hepatozoon*

## Abstract

**Background:**

Population of stray dogs is significant in large cities of Nepal, such as Kathmandu. Most of stray dogs suffer a lack of basic health care. Considering the clinical relevance, the broad distribution and the lack of information of canine vector borne diseases (CVBD) in Nepal, the aim of this study was to evaluate the prevalence of different vector-borne pathogens (VBP) in stray dogs living in the metropolitan area of Kathmandu, and to assess different traits as possible risk factors.

**Methods:**

A total of 70 canine blood samples from stray dogs attended at the Kathmandu Animal Treatment Centre during August 2017 were collected on filter paper (Flinders Technology Associates (FTA) cards). Data regarding signalment, clinical signs and epidemiological characteristics were recorded for each animal. Real-time polymerase chain reaction assays were performed for *Leishmania* spp., *Ehrlichia* spp.*/Anaplasma* spp., *Babesia* spp.*/Theileria* spp. and *Hepatozoon canis*.

**Results:**

The overall prevalence detected was 31.43% for *Hepatozoon canis*, 31.43% for *Anaplasma platys*, 27.14% for *Ehrlichia canis*, 18.57% for *Leishmania donovani* species complex, 12.86% for isolates corresponding to *Theileria* spp., 12.86% for *Babesia vogeli* and 2.86% for *B. gibsoni.* A total of 81.43% of the dogs were positive to at least one of the VBP tested. Co-infections were detected in 41.43% of the dogs. Dogs positive to any of the VBP tested, and particularly to *E. canis*, were older than those that were negative.

**Conclusions:**

To our knowledge, this is the first molecular detection of VBP in stray dogs from Kathmandu, Nepal. The high prevalence of VBP detected highlights the need to implement a surveillance programme and control strategies for these CVBD in the population of stray dogs in this area.
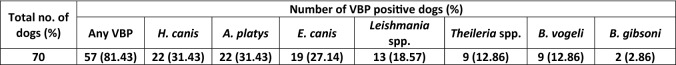

## Background

Populations of stray dogs are significant in large cities of Nepal, such as Kathmandu [1]. However, data on the demographics of stray dogs in Nepal remains unknown [2]. Most of free-roaming dogs suffer from malnutrition, diseases and lack of basic health care (deworming, antiparasitic treatments or vaccination) [2].

While the status of rabies in these dog populations is a major issue for the Public Health in Nepal [3], information about the prevalence of other zoonotic diseases [2, 4, 5] and specifically, canine vector borne diseases (CVBD) is really scarce. A molecular evaluation of different vector-borne pathogens (VBP) is lacking.

Kathmandu Valley is located in the warm temperate area of the country, where the climate is fairly temperate, and appropiate for the presence of different vectors of relevance for the dog population such as sand flies and hard ticks.

The aim of this study was to evaluate the prevalence of different VBP in stray dogs living in the metropolitan area of Kathmandu, Nepal, and to assess different traits as possible risk factors. Taking into account the clinical relevance, the broad distribution and the lack of information in dogs from Nepal, the pathogens evaluated in this study were *Leishmania* spp. *Ehrlichia* spp./*Anaplasma* spp., *Babesia* spp./*Theileria* spp. and *Hepatozoon canis*.

## Methods

Canine blood samples from stray dogs attending the Kathmandu Animal Treatment Centre during August 2017 were collected for further molecular analyses. Different data regarding signalment, clinical signs, and epidemiological characteristics were recorded for each animal. We aimed to perform real-time polymerase chain reaction assays for *Leishmania* spp., *Ehrlichia* spp./*Anaplasma* spp., *Babesia* spp.*/Theileria* spp. and *Hepatozoon canis*.

### Animals and samples, and data collection

A total of 70 blood samples from stray dogs attended at the Kathmandu Animal Treatment Centre (KAT Centre) during August 2017 were collected from the core of the metropolitan area of Kathmandu, Nepal. Stray dogs were opportunistically sampled when they were brought into the clinic for medical treatment or for neutering procedures as a part of animal birth control (ABC) programmes. Dogs with and without clinical signs were included in this study. Dog blood samples obtained by venipuncture were spotted on Whatman FTA® (Flinders Technology Associates) classic cards (Whatman International Ltd, Maidstone, UK) for further molecular analyses. To avoid cross-contamination, these samples were stored in separate plastic bags that included storage desiccant packets to ensure that FTA cards remained dry during transport and storage. Samples were kept at room temperature during the entire campaign and were later stored at − 20 °C until DNA extraction. Data regarding signalment, clinical signs and presence of ectoparasites were recorded for each animal.

### DNA extraction and quality assessment

DNA was extracted using SpeedTools Tissue DNA extraction commercial kit (Biotools, B&M Laboratories, S.A., Madrid, Spain) according to the manufacturer’s instructions. All DNA samples were stored at − 20 °C until use. The extraction yield (quality and quantity of the extracted DNA) was assessed by means of spectrophotometry (Nanodrop^TM^; Thermo Fisher Scientific, Waltham, USA). The eukaryotic *18S* ribosomal ribonucleic acid (*18S* rRNA) (Thermo Fisher Scientific) was used as an internal reference of canine genomic DNA to ensure proper extraction (presence/absence of DNA inhibition factors).

### Real-time PCR assays

Real-time PCR assays were used to target selected species of VBP, including *Babesia/Theileria* species, *Ehrlichia/Anaplasma* species, *Hepatozoon canis* and *Leishmania donovani* complex species as previously described [6, 7].

Real-time PCR reactions were conducted in a 20 µl reaction mixture containing PowerUp SYBR Green master mix (Thermo Fisher Scientific, Carlsbad, CA, USA), specific primers (Table 1) and 4 µl of 1/5 diluted DNA. The thermal cycling profile was 50 °C for 2 min and 95 °C for 2 min, followed by 40 cycles at 95 °C for 3 s and 60 °C for 30 s and a dissociation curve added at the end of the run to asses PCR-specificity. Commercial DNA and water were used with each amplification run as a positive and negative PCR controls, respectively. Positive-PCR amplicons were directly sequenced to characterize pathogens at the species level using the same primers. Subsequent sequencing was performed in all the positive samples, using the Big Dye® Terminator version 3.1 Cycle Sequencing kit (Thermo Fisher Scientific, Carlsbad, CA, USA) following the manufacturer’s instructions. Sequences obtained were compared with those deposited on GenBank using the Basic Local Alignment Search Tool (BLAST).

**Table 1:** PCR primers used for detection of selected vector-borne pathogens: *Ehrlichia* spp., *Anaplasma* spp., *Hepatozoon canis, Babesia* spp., *Theileria* spp. and *Leishmania* spp. in dogs from Kathmandu, Nepal

Species	Target gene	Forward primer (5’-3’)	Reverse primer (5’- 3’)	References
*Ehrlichia* spp.; *Anaplasma* spp.	*16S* rRNA	GCAAGCYTAACACATGCAAGTCG	GGATTATACAGTATTACCCAYCATTTCTARTG	[6]
*Hepatozoon canis*	*18S* rRNA	CTTACCGTGGCAGTGACGGT	ATTGTTATTTCTTGTTACTACCTCTCTCAAAC	
Piroplasmida (*Babesia/Theileria* spp.)		GACGATCAGATACCGTCGTAGTCC	CAGAACCCAAAGACTTTGATTTCTCTC	
*Leishmania* spp.	Kinetoplast minicircle DNA	AACTTTTCTGGTCCTCCGGGTAG	ACCCCCAGTTTCCCGCC	[7]

*Abbreviation*: rRNA ribosomal ribonucleic acid

### Statistical analysis

The results were statistically analysed using the software SAS, version 9.4 (SAS Institute, Cary, NC, USA). Statistical associations between VBP and epidemiological data recorded for each stray dog were analysed using Chi-square test or Fisherʼs exact test, where appropriate, and Studentʼs t-test to evaluate mean age with positive or negative effects for different vector-borne pathogens. In order to exclude possible confounding factors, complementary logistic regression analysis with backward elimination was performed with those variables that showed a statistical association. The level of statistical significance was established at *P* < 0.05.

## Results

### Dog population description

Samples were collected from 24 different locations of the metropolitan area of Kathmandu, Nepal: Balaju (*n* = 2); Bansbari (*n* = 2); Budanilkantha (*n* = 25); Chapali Bhadrakali (*n* = 3); Dhapasi (*n* = 1); Dhumbarahi (*n* = 2); Ring Road Dhungedara, Banasthali (*n* = 3); Durbar Marg (*n* = 1), Gaurighat (*n* = 1); Golfutar (*n* = 3); Hattigauda (*n* = 1); Jamal (*n* = 3); Kalimati (*n* = 2); Lazimpat (*n* = 2); Lyangfang (*n* = 1); Maharajgunj (*n* = 3); Manamaiju (*n* = 1); Mandikhatar (*n* = 3); Nayabazar (*n* = 2); New Road (*n* = 1); Shovabhagbati (*n* = 1); Swoyambhu (*n* = 4); Thulo Bharyang (*n* = 2); and Tokha (*n* = 1).

Among the 70 stray dogs included in the study, 29 (41.43%; 95% CI: 29.9–53%) were males and 41 (58.57%; 95% CI: 47.0–70.1%) were females. Most of the dogs were local mongrels that could not be associated with any breed (97.14%; 95% CI: 93.2–100%) except for two Japanese Spitz dogs. Age of the dogs ranged from one month to 14 years, with a mean age of 3.78 ± 2.93 years–old. A total of 29/70 (41.43%; 95% CI: 29.9–53.0%) were neutered.

Considering the clinical status, a total of 46/70 dogs (65.71%; 95% CI: 54.6–75.8%) presented clinical signs, and 24/70 (34.39%; 95% CI: 23.2–45.4%) remained apparently healthy. Regarding physical examination, only two dogs were dehydrated, and two presented lymphadenopathies. When considering mucous membrane examination, pallor was detected in eight dogs (11.43%; 95% CI: 4.0–18.9%), and capillary refill time (CRT) was longer than two seconds in three dogs (4.29%; 95% CI: 0–9.0%). Thirty four out of 70 of the dogs (48.57%; 95% CI: 36.9–60.3%) presented underweight (BCS ≤ 4/9). On the other hand, only 13 dogs (18.57%, 95% CI: 9.5–27.7%) presented overweight (BCS = 6/9; *n* = 6 and BCS =7/9; *n* = 7).

When assessing ectoparasites presence, hard ticks (family Ixodidae) were detected on 42 out of 70 dogs (60%; 95% CI: 48.5–71.5%). No soft ticks (family Argasidae) were detected. Furthermore, 31 out of 70 (44.29%; 95% CI: 32.6–55.9%) had flea infestation. A total of 24 out of 70 (34.28%; 95% CI: 23.2–45.4%) carried both ectoparasites. On the other hand, skin lesions compatible with mange were present in 23 dogs (32.86%; 95% CI: 21.9–43.9%). Concurrent ticks, fleas and mange-suspected infestation were presented in eight dogs (11.42%; 95% CI: 4.0–18.9%). All data regarding signalment, clinical, and environmental variables are shown in Table 2.

**Table 2:** Comparison of prevalence of selected VBP in association with different epidemiological data recorded in the study

Variable	Total no. of dogs (%)	Number of VBP positive dogs (%)							
		Any VBP	*H. canis*	*A. platys*	*E. canis*	*Leishmania* spp.	*Theileria* spp.	*B. vogeli*	*B. gibsoni*
	70	57 (81.43)	22 (31.43)	22 (31.43)	19 (27.14)	13 (18.57)	9 (12.86)	9 (12.86)	2 (2.86)
Average age	70								
Positive	na	4.09 ± 3.30*	3.96 ± 2.57	3.77 ± 2.76	5.16 ± 3.39*	4.40 ± 2.69	3.51 ± 3.00	2.42 ± 2.05	1.75 ± 0.35
Negative	na	2.40 ± 1.09	3.7 ± 3.10	3.79 ± 3.03	3.27 ± 2.59	3.64 ± 2.98	3.82 ± 2.94	3.98 ± 3.00	3.84 ± 2.95
Sex	70								
Male	29 (41.43)	25 (86.21)	12 (41.38)	9 (31.03)	11 (37.93)	3 (10.34)	4 (13.79)	2 (6.90)	1 (3.45)
Female	41 (58.57)	32 (78.05)	10 (24.39)	13 (31.71)	8 (19.51)	10 (24.39)	5 (12.20)	7 (17.07)	1 (2.44)
Age	70								
Puppy (<1-year-old)	8 (11.43)	5 (62.50)	1 (12.50)	2 (25.00)	0 (0)	1 (12.50)	3 (37.50)*	1 (12.50)	0 (0)
Adult (1–5-year-old)	42 (60.00)	34 (80.95)	15 (35.71)	13 (30.95)	12 (28.57)	6 (14.29)	3 (7.14)	7 (16.67)	2 (4.76)
Mature adult (>5-year-old)	20 (28.57)	18 (90.00)	6 (30.00)	7 (35.00)	7 (35)	6 (30.00)	3 (15.00)	1 (5.00)	0 (0)
Spay/neutered	70								
Yes	29 (41.43)	26 (89.66)	10 (34.48)	8 (27.59)	12 (41.38)*	7 (24.14)	3 (10.34)	2 (6.90)	1 (3.45)
No	41 (58.57)	31 (75.61)	12 (29.27)	14 (34.15)	7 (17.07)	6 (14.63)	6 (14.63)	7 (17.07)	1 (2.44)
BCS	70								
Underweight (1–< 5)	34 (48.57)	26 (76.47)	10 (29.41)	8 (23.53)	10 (29.41)	6 (17.65)	3 (8.82)	6 (17.65)	0 (0)
Normal weight (5)	23 (32.86)	18 (78.26)	9 (39.13)	9 (39.13)	5 (21.74)	3 (13.04)	3 (13.04)	2 (8.70)	2 (8.70)
Overweight (> 5–9)	13 (18.57)	13 (100)	3 (23.08)	5 (38.46)	4 (30.77)	4 (30.77)	3 (23.08)	1 (7.69)	0 (0)
Clinical signs	70								
Yes	46 (65.71)	39 (84.71)	15 (32.61)	14 (30.43)	15 (32.61)	8 (17.39)	6 (13.04)	5 (10.87)	1 (2.17)
No	24 (34.39)	18 (75.00)	7 (29.17)	8 (33.33)	4 (16.67)	5 (20.83)	3 (12.50)	4 (16.67)	1 (4.17)
Mucous membrane	70								
Normal	62 (88.57)	51 (82.26)	21 (33.87)	19 (30.65)	17 (27.42)	12 (19.35)	7 (11.29)	8 (12.90)	2 (3.23)
Pallor	8 (11.43)	6 (75.00)	1 (12.50)	3 (37.50)	2 (25.00)	1 (12.50)	2 (25.00)	1 (12.50)	0 (0)
CRT	70								
Normal CRT	67 (95.71)	54 (80.60)	21 (31.34)	21 (31.34)	18 (26.87)	12 (17.91)	9 (13.43)	9 (13.43)	2 (2.99)
Longer than 2 sec	3 (4.29)	3 (100)	1 (33.33)	1 (33.33)	1 (33.33)	1 (33.33)	0 (0)	0 (0)	0 (0)
Dehydration	70								
Yes	2 (2.86)	2 (100)	0 (0)	0 (0)	1 (50.00)	1 (50.00)	0 (0)	0 (0)	0 (0)
No	68 (97.14)	55 (80.88)	22 (32.35)	22 (32.35)	18 (26.47)	12 (17.65)	9 (13.24)	9 (13.24)	2 (2.94)
Lymphadenopathies	70								
Yes	2 (2.86)	2 (100)	0 (0)	1 (50.00)	1 (50.00)	0 (0)	1 (50.00)	0 (0)	0 (0)
No	68 (97.14)	55 (80.88)	22 (32.35)	21 (30.88)	18 (26.47)	13 (19.12)	8 (11.76)	9 (13.24)	2 (2.94)
Tick infestation	70								
Yes	42 (60.00)	37 (88.10)	15 (35.71)	14 (33.33)	13 (30.95)	5 (11.90)	7 (16.67)	4 (9.52)	2 (4.76)
No	28 (40.00)	20 (71.43)	7 (25.00)	8 (28.57)	6 (21.43)	8 (28.57)	2 (7.14)	5 (17.86)	0 (0)
Flea infestation	70								
Yes	31 (44.29)	25 (80.65)	7 (22.58)	13 (41.94)	6 (19.35)	5 (16.13)	4 (12.90)	5 (16.13)	0 (0)
No	39 (55.71)	32 (82.05)	15 (38.46)	9 (23.08)	13 (33.33)	8 (20.51)	5 (12.82)	4 (10.26)	2 (5.13)
Mange	70								
Yes	23 (32.86)	19 (82.61)	9 (39.13)	7 (30.43)	7 (30.43)	4 (17.39)	4 (17.39)	4 (17.39)	0 (0)
No	47 (67.14)	38 (80.85)	13 (27.66)	15 (31.91)	12 (25.53)	9 (19.15)	5 (10.64)	5 (10.64)	2 (4.26)

*Abbreviations*: VBP, vector-borne pathogens; *H. canis*, *Hepatozoon canis*; *A. platys*, *Anaplasma platys*; *E. canis*, *Ehrlichia canis*; *Leishmania* spp., *Leishmania donovani* species complex; *B. canis vogeli*, *Babesia canis vogeli*; *B. gibsoni*, *Babesia gibsoni*; BCS, body condition score; CRT, capillary refill time; na, not applicable

*P < 0.05

### PCR amplification and sequencing results

*Hepatozoon canis* was detected in 22 (31.43%; 95% CI: 20.6–42.2%) of the samples using a species-specific PCR. A total of 41 dogs (58.57%; 95% CI: 47.0–70.1%) were positive to *Anaplasma/Ehrlichia* spp., with 22 PCR sequences (31.43%; 95% CI: 20.6–42.3%) corresponding to *Anaplasma platys*, and 19 (27.14%; 95% CI: 16.7–37.6%) to *Ehrlichia canis. Leishmania donovani* complex species were detected in 13 dogs (18.57%; 95% CI: 9.5–27.7%).

A total of 20 dogs (28.57%; 95% CI: 18.0–39.2%) were positive to *Babesia* spp./*Theileria* spp. These values correspond to a total rate of 15.71% (11/70; CI: 7.2–24.2%) of *Babesia* spp. further determined as 12.86% (9/70; 95% CI:5.0–20.7%) for *B. vogeli* and 2.86% (2/70; 95% CI: 0–6.8%) for *B. gibsoni*, and 12.86% (9/70; 95% CI: 5.0–20.7%) to an unidentified species that matched with several isolates of *Theileria* spp. (*T. sinensis, T. luwenshuni* or *T. annulata* among others) but unrelated to *Babesia vulpes* (formerly known as *Theileria annae*). The prevalence of CVBD agents according to different variables, including signalment, clinical findings, and environmental data are shown in Table 2.

When considering co-infections, 40% (28/70; 95% CI: 28.5–51.1%) of dogs were positive to single VBP, and 41.43% (29/70; 95% CI: 29.9–53.0%) presented co-infections. Of these, nine dogs (12.86%; 95% CI: 5.0–20.7%) were positive to three different VBP (Table 3). Considering single infections, *E. canis* was the most detected VBP (9/70; 12.86%; 95% CI: 5.0–20.7%) and *B. vogeli* was found in only two dogs (2.86%, 95% CI: 0–6.8%). *Babesia gibsoni* was always found in combination with other VBP infection. The most common co-infections detected in the study were *H. canis* + *E. canis* (7/70; 10%; 95% CI: 3.0–17.0%) and *H. canis* + *A. platys* (6/70; 8.57%; 95% CI: 2.0–15.1%). Data regarding single infections and co-infections are shown in Table 3.

**Table 3:** Single infections and co-infections for the VBP studied in stray dogs from Kathmandu, Nepal

Vector-borne pathogen		No. of PCR-positive dogs (%)	95% CI
Single infections		28 (40.00)	28.5–51.5
*Ehrlichia canis*		9 (12.86)	5.0–20.7
*Anaplasma platys*		7 (10.00)	3.0–17.0
*Hepatozoon canis*		5 (7.14)	1.1–13.2
*Leishmania donovani* species complex		4 (5.71)	0.3–11.2
*Babesia vogeli*		2 (2.86)	0–6.8
*Theileria* spp.^a^		1 (1.43)	0–4.2
Co-infections		29 (41.43)	29.9–53.0
Co-infection (2 VBP)	*E. canis + H. canis*	4 (5.71)	0.3–11.2
	*A. platys* + *H. canis*	4 (5.71)	0.3–11.2
	*A. platys* + *Theileria* spp.	3 (4.28)	0–9.0
	*B. gibsoni* + *A. platys*	2 (2.86)	0–6.8
	*L. donovani* complex + *E. canis*	2 (2.86)	0–6.8
	*L. donovani complex + H. canis*	1 (1.43)	0–4.2
	*L. donovani* complex + A. *platys*	1 (1.43)	0–4.2
	*B. vogeli* + *L. donovani* complex	1 (1.43)	0–4.2
	*Theileria* spp. + *H. canis*	1 (1.43)	0–4.2
	*A. platys* + *B. vogeli*	1 (1.43)	0–4.2
Co-infection (3 VBP)	*Theileria* spp. + *A. platys* + *H. canis*	2 (2.86)	0–6.8
	*B. vogeli* + *E. canis* + *H. canis*	2 (2.86)	0–6.8
	*B. vogeli* + *E. canis* + *L. donovani* complex	1 (1.43)	0–4.2
	*B. gibsoni* + *E. canis* + *H. canis*	1 (1.43)	0–4.2
	*B. vogeli* + *A. platys* + *L. donovani* complex	1 (1.43)	0–4.2
	*Theileria* spp. + *H. canis* + *L. donovani* complex	1 (1.43)	0–4.2
	*Theileria* spp. + *A. platys* + *L. donovani* complex	1 (1.43)	0–4.2
Total		57 (81.43)	72.3–90.5

^a^*Theileria* spp.: sequences compatible with *Theileria* spp. (sequences were not further confirmed by amplification of the long *18S* sequence)

Abbreviation: CI, confidence interval

### Epidemiological and clinical data

We did not detect statistically significant associations between the presence of VBP studied and sex (*χ*^2^ = 0.74, *df =* 1, *P* = 0.38) or BCS (*χ*^2^ = 3.67, *df =* 2, *P* = 0.16). Considering any VBP infection, positive dogs were older (4.09 ± 3.3 years-old) than negative dogs (2.4 ± 1.09 years-old) (Studentʼs t-test, *t* = 1.92, *P =* 0.05). Furthermore, there was no relationship between the presence of clinical signs and the infection with any VBP (*χ*^2^ = 0.99, *df =* 1, *P* = 0.31) **(**Table 2).

The co-infection with more than one VBP in the same animal was not associated with any of the variables evaluated. Additionally, tick-infestation detected during physical exam of dogs was not statistically associated with any of the tick-vector borne agents studied (*Hepatozoon canis* (*χ*^2^ = 0.89, *df =* 1, *P* = 0.34), *A. platys* (*χ*^2^ = 0.18, *df =* 1, *P* = 0.67), *E. canis* (*χ*^2^ = 0.77, *df =* 1, *P* = 0.38), or any *Babesia/Theileria* spp. (*χ*^2^ = 1.16, *df =* 1, *P* = 0.28). This only suggests the unreliability of tick detection by clinicians in the moment of hospital application as suggestive of tick-borne agents.

*Ehrlichia canis* was the only pathogen displaying a statistical association with dog-derived traits. When looking to *E. canis* infection specifically, it was observed that dogs with a positive PCR result were also older (5.16 ± 3.39 years-old) than negative dogs (3.27 ± 2.59 years-old) (Studentʼs t-test, *t* = 2.49, *P =* 0.0153). On the other hand, neutered status was also associated with PCR-positive result for *E. canis* (*χ*^2^ = 5.07, *df =* 1, *P* = 0.024). However, complementary logistic regression analysis with backward elimination was performed including those variables in a model and demonstrated that these associations were not statistically significant (neutered status (*P* = 0.09; OR: 2.68; 95% CI: 0.85–8.43) and age (*P =* 0.067; OR: 1.2; 95% CI: 0.99–1.47).

## Discussion

To our knowledge, this study addressed the first molecular survey of VBP in stray dogs from the metropolitan area of Kathmandu, Nepal, and the first report of *H. canis* infection in dogs from this region. A high prevalence of VBP was detected in this study using real-time PCR technique. The results presented here support the scarce findings of previous studies that detected *Leishmania* spp. in dogs in some regions of Nepal [8]. *Babesia* sp., *Ehrlichia* sp. and *Anaplasma* sp. have also been recently detected by microscopy in blood smears from hyperthermic owned dogs in Kathmandu Valley [9, 10].

Previous studies in the region have found an overall prevalence of haemoparasites between 10–17.14% in dogs considered under risk based on clinical signs like fever or hyperthermic dogs, or those carrying tick-infestation [9]. However, our study shows a higher prevalence of selected VBP (81.43%). This could be explained because previous studies were performed on owned dogs (that usually receive the adequate veterinarian care). Most of the stray dogs involved in our study were highly exposed to tick and sand fly bites. In addition, differences in the techniques employed for the detection of these pathogens could explain, at least partially, the differences in the prevalences detected. It has been previously established that real time-PCR is considerably more sensitive than visualization of the VBP in a blood smear, especially in those cases of low burden of VBP [11–14].

*Hepatozoon canis* and *A. platys* were the most frequently detected agents. *Hepatozoon canis* has been recently detected in dogs from Northeast India with a prevalence of 38% [15], slightly higher than the prevalence described here (31.43%). These apicomplexan protozoans are transmitted by the ingestion of an infected tick [16]. The presence of ticks on dogs is sometimes used in practice as a predictor of some CVBD, especially in the lack of specific diagnostic tests. However, this study shows the lack of significant association between *H. canis* infection and detection of ticks in dogs during the physical exam (*P =* 0.34).

Sequencing confirmed that 31.43% of the dogs were positive to *A. platys,* and 27.14% to *E. canis.* Both rickettsial agents are transmitted by the same vector, *Rhipicephalus sanguineus* (*sensu lato*). As previously described for *H. canis*, tick infestation was detected in a high percentage of dogs infected by *A. platys* or *E. canis* (63.63% and 68.42%, respectively). Previous studies showed high prevalence of ectoparasite infestation in dogs from this area [2], and a recent review did show that a wide variety of tick species are parasites of dogs in Nepal [17].

Infections with *Anaplasma* spp. (1.34%) and *Ehrlichia* spp. (10.66%) have been recently detected in blood smears from hyperthermic dogs in Kathmandu [10]. The dogs infected with *E. canis* were older than the negative dogs in our study, probably due to a higher exposure to the vectors and their pathogens. *Ehrlichia canis* infection was more frequently detected in male dogs, although statistical test was non-significant (*P =* 0.08). This could be related to behavioural characteristics, as previously suggested [18].

Nepal is recognized as endemic for *L. donovani* [19]. However, the role of the dog as a reservoir in the cycle of this anthroponotic visceral leishmaniasis (AVL) (also known as kala-azar) and transmitted by *Phlebotomus argentipes* (anthropophilic vector) remains unknown. Some studies in India have hypothesized that dogs may constitute a reservoir for this species of *Leishmania* [20]. A previous study performed in the Terai region (southern Nepal) has shown that *P. argentipes* and *P. papatasi* with predilection to feed on humans or cattle, also feed on dog blood [8]. *Phlebotomus papatasi* has not been incriminated as a vector for *L. donovani* [8, 21]. However, this sand fly is a reported vector for other *Leishmania* spp. (*L. major* and *L. infantum*) in the Old World, suggesting that Nepal could become endemic for zoonotic leishmaniosis [22]. Our study shows a prevalence of 18.57% of *Leishmania donovani* complex in the tested stray dogs. There was no statistically significant correlation between *Leishmania* spp. detection and any signalment data, clinical findings, and environmental data. It was not possible to establish if these animals were suffering a clinical presentation of leishmaniosis or were just infected and carrying the pathogen at a low burden. The lack of compatible clinical signs and the low number of copies of parasite DNA detected, could support the second hypothesis. Thus, having confirmed the presence of *Leishmania* spp. by molecular tests, it would be interesting in the future to establish the role of the dog in the human leishmaniosis in Kathmandu.

Canine babesiosis has been recently reported in dogs from Kathmandu [9] and *B. gibsoni* constituted the most detected vector-borne agent found in dogs from Northeast India (43% of the dogs positive to *B. gibsoni* and 3% positive to *B. vogeli*) [15]. In contrast, in our study, prevalence was higher for *B. vogeli* (12.86%), and lower for *B. gibsoni* (2.86%). *Babesia gibsoni* has been widely described in Asia [15] and is transmitted by *Haemaphysalis longicornis* [23]. Other species of the genus *Haemaphysalis* have been previously detected in Nepal [17]. On the other hand, the main vectors described for *B. vogeli* are ticks from different genera (*Dermacentor* spp., *Ixodes* spp., and *R. sanguineus* (*s.l.*)) also found in dogs from Nepal [17]. The authors of the present study are cautious with the DNA sequences of *Theileria* spp. detected in dogs.

An unexpected finding from this study was the high rate of co-infections, detected in 29 out of 70 dogs (41.43%). In agreement with this finding, another recent study in India has shown a high prevalence of dogs presenting co-infection by two or more agents (44/130, 34%) [15]. The most common co-infection detected in our study was *H. canis + E. canis.* Canine hepatozoonosis and ehrlichiosis are both tick-borne diseases transmitted by *R. sanguineus* (*s.l.*). Some interactions between the pathogens and host cells in this co-infection have been previously suggested [24]. The high ratio of co-infections could be explained by the interactions of some of these pathogens when infecting dogs, as previously described [25].

There was no effect of BCS or sex on the prevalence of these VBP. The animals included in the study were stray dogs with difficulties to feed properly and most of them presented a low BCS. This could be related to the lack of statistical differences regardless of whether they present clinical signs. A previous study in Nepal detected that most of the dogs (80%; *n* = 47) had an adequate BCS. However, ecto- and endoparasites were also detected in most of them (83%; *n* = 49) [2].The lack of association between the presence of infection and the sex of the dogs in this study is consistent with other studies evaluating VBP in dogs from India [15, 26] and from Kathmandu Valley, Nepal [9, 10].

Clinical findings detected in the dogs included in the study were not directly related with the classical clinical picture of CVBD, and consisted mainly on wounds, myiasis, bone fractures, fungal skin lesions, and limb loss, among others. The lack of clinical signs in asymptomatic carriers, the co-infection with multiple VBP in the same animal or even the poor health condition in non-infected dogs constitute one of the barriers in the clinical diagnosis approach for these CVBD. In agreement with our work, a previous study performed in Chitwan District (central Nepal), also showed that there was a considerable number of apparently healthy dogs carrying haemoprotozoans and ectoparasites (49/59; 83%) [2].

Future studies should focus on the evaluation of possible risk factors, as well as the presence of competent arthropod vectors to understand biological cycles of the parasites, the role of the dog as a possible reservoir in zoonosis transmission, and the control of these VBP affecting dog populations and, potentially, the human population.

## Conclusions

To our knowledge, this study constitutes the first molecular report of VBP in stray dogs from Kathmandu, Nepal. Our results highlight the importance of several CVBD that should not be underestimated in the metropolitan area of Kathmandu. The prevention and treatment of these CVBD must be taken under consideration and implemented in the veterinary control in concurrence with population awareness programmes, animal birth control or rabies vaccination programmes. Further on this, considering the high detected prevalence of VBP and following the WSAVA vaccination recommendations, a check-up including ecto- and endoparasite control should be performed prior to vaccination in apparently healthy dogs that could be potentially infected. Further studies are needed to understand the role of the dogs and arthropod vectors in the transmission of these and other VBP in the metropolitan area of Kathmandu.

## Data Availability

All data generated or analysed during this study are included in this published article.

## Abbreviations

BCS: body condition score
CRT: capillary refill time
CVBD: canine vector-borne disease
na: not applicable
rRNA: ribosomal ribonucleic acid
VBP: vector-borne pathogen
